# Experience with 808-nm diode laser in the treatment of 47 cases of oral vascular anomalies

**DOI:** 10.1590/1807-3107bor-2024.vol38.0025

**Published:** 2024-04-05

**Authors:** Fernanda Vieira HEIMLICH, José Alcides Almeida de ARRUDA, Camila de Nazaré Alves de Oliveira KATO, Leni Verônica de Oliveira SILVA, Leandro Napier SOUZA, Marcus Vinicius Lucas FERREIRA, João de Jesus Viana PINHEIRO, Tarcília Aparecida SILVA, Lucas Guimarães ABREU, Ricardo Alves MESQUITA

**Affiliations:** (a) Department of Oral Surgery, Pathology and Clinical Dentistry, School of Dentistry, Universidade Federal de Minas Gerais, Belo Horizonte, Minas Gerais, Brazil.; (b) Department of Oral Surgery, Pathology and Clinical Dentistry, School of Dentistry, Universidade Federal de Minas Gerais, Belo Horizonte, Minas Gerais, Brazil.; (c) Department of Oral Surgery, Pathology and Clinical Dentistry, School of Dentistry, Universidade Federal de Minas Gerais, Belo Horizonte, Minas Gerais, Brazil.; (d) Department of Oral Surgery, Pathology and Clinical Dentistry, School of Dentistry, Universidade Federal de Minas Gerais, Belo Horizonte, Minas Gerais, Brazil.; (e) Department of Oral Surgery, Pathology and Clinical Dentistry, School of Dentistry, Universidade Federal de Minas Gerais, Belo Horizonte, Minas Gerais, Brazil.; (f) Department of Restorative Dentistry, School of Dentistry, Universidade Federal de Minas Gerais, Belo Horizonte, Minas Gerais, Brazil.; (g) Cell Cultivation Laboratory, Universidade Federal do Pará, Belém, Pará, Brazil.; (h) Department of Oral Surgery, Pathology and Clinical Dentistry, School of Dentistry, Universidade Federal de Minas Gerais, Belo Horizonte, Minas Gerais, Brazil.; (i) Department of Child and Adolescent Oral Health, School of Dentistry, Universidade Federal de Minas Gerais, Belo Horizonte, Minas Gerais, Brazil.; (j) Department of Oral Surgery, Pathology and Clinical Dentistry, School of Dentistry, Universidade Federal de Minas Gerais, Belo Horizonte, Minas Gerais, Brazil.

**Keywords:** diode laser, oral cavity, oral medicine, photocoagulation, vascular anomalies

## Abstract

Treatment of oral vascular anomalies (OVA) has focused on minimally invasive techniques rather than radical surgery. We investigated the efficacy and safety of diode laser using the photocoagulation technique in the management of OVA. Forty-seven subjects with OVA were treated with forced dehydration with induced photocoagulation (FDIP) using diode laser (808 nm/4.5 W). This series consisted mostly of male (63.8%) and non-white (63.8%) patients with a mean age of 57.4 years. Varices (91.5%), venous malformations (6.4%), and hemangiomas (2.1%) with a mean size of 7.1 (±4.9) mm were the conditions treated. OVA presented as a nodular lesion (63.8%) involving mainly the lower lip (46.8%). Pulsed laser mode was used as standard and the number of applications varied from one to four sessions, with the majority requiring only one (83%) FDIP session. Kaplan-Meier analysis revealed that complete clinical healing can occur on the 15th day (*n=*9/29.5%), followed by the 20th (*n=*6/45.5%), and 30th (*n=*7/70.5%) days. Postoperative edema was observed in 31 (66%) patients, and recurrence of the lesion occurred in two (4.2%). Based on the data on complete clinical healing, minimal patient discomfort, and satisfactory esthetic results, we can confirm that FDIP by diode laser is a promising candidate for the safe and efficacious treatment of OVA.

## Introduction

Vascular anomalies are endothelial conditions that can affect capillaries, arteries, veins, and lymphatic vessels.^1^ According to the International Society for the Study of Vascular Abnormalities^1,2^ based on the seminal report by Mulliken and Glowacki^3^ vascular anomalies are currently classified into two main types: vascular tumors and vascular malformations. Vascular anomalies occur in a wide range of age groups and affect various organs, including the oral cavity.^4,5^ Individuals with vascular anomalies in the oral cavity may experience pain, bleeding, and functional and/or esthetic complaints. Furthermore, an increased susceptibility to trauma and the resulting deleterious implications has been documented; the treatment of these conditions is therefore recommended.^5^

In recent years, more emphasis has been placed on minimally invasive techniques due to the potential morbidities of radical surgery due to the possible morbidities of the latter in the management of vascular anomalies.^6^ While surgical intervention remains a highly valued option for selected patients, other therapies such as local/systemic corticosteroids, sclerotherapy, and more recently, forced dehydration with induced photocoagulation (FDIP) using laser are also used.^4,5,7^ In FDIP, the diode laser is useful because it is highly absorbed by chromophores, such as hemoglobin, melanin, and collagen, and because it can cut and coagulate soft tissue, providing hemostasis and efficient tissue ablation.^8,9^ Once the laser has penetrated the tissue, it generates heat with coagulation capacity at a depth of 7.0 to 10.0 mm, a process called photocoagulation.^10,11^ In this process, energy is released through an optical fiber, maintained between 2.0 and 3.0 mm without contact with the target tissue.^10^

FDIP using diode laser has become an effective, reliable, and bloodless therapeutic option for patients with oral vascular anomalies (OVA).^8,10-12^ Moreover, the possibility of recurrence, damage to tissue adjacent to the lesion, and scar tissue formation are reduced compared to surgery with scalpel.^13,14^ Since the vast majority of OVA are small and of the low-flow type, treatment with FDIP can provide satisfactory and promising results. However, the application of this technique in the treatment of OVA is little explored in the literature, especially with regard to long-term follow-up and recurrence data.^8,10^ The purpose of the present study was to evaluate the efficacy and safety of FDIP by diode laser in the management of OVA.

## Methodology

### Study design, setting, and ethical issues

In the present series, 47 cases of OVA were included as a convenience sample. All patients were recruited and treated consecutively at the referral service of the Oral Medicine at the School of Dentistry of the Universidade Federal de Minas Gerais from 2016 to 2018. The Strengthening the Reporting of Observational Studies in Epidemiology (STROBE) guidelines were followed.^15^ The study was approved by the Ethics Committee on Human Research of the Institution (No. 61214916.9.0000.5149) and the participants agreed with the publication of their cases in accordance with the Declaration of Helsinki.

### Diagnostic rendering, patients, and trans- and postoperative assessments

The diagnosis of OVA was based on the ISSVA classification,^16^ considering the clinical characteristics of the lesion and diascopy findings. Cases of varicose veins/varix, hemangiomas, and venous malformations were considered. Clinically, oral varicose vein/varix present as single or multiple irregular papules or nodules, are asymptomatic and blue-purple in color, and mainly affect older patients.^17^ Oral hemangioma (capillary form) present as flat red or bluish-purple macules, papules or nodules, are smooth or lobulated, and contain numerous small capillaries.^18^ Venous malformations are defined as isolated, irregular, superficial, purplish-blue nodules that are easily compressible.^3^ In the latter two diagnoses, the patient had a history of congenital or acquired lesions in the first years of life.^19^ The inclusion criteria were patients with OVA whose chief complaints were related to esthetic and/or functional aspects, and that such manifestations were clinically superficial and of slow flow. Patients who refused management with the therapy of interest, those who had undergone another therapy (e.g., sclerotherapy), or who did adhered to follow-up guidelines, and those with severe systemic conditions were excluded from the study. Of note, none of the patients withdrew from the study before or during data collection.

**Figure 1:**
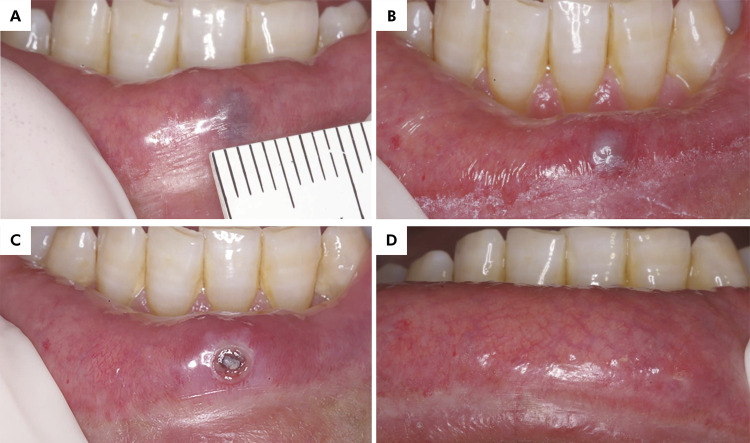
A 50-year-old male patient with oral varix in the lower lip. (A) Purplish well-delimited papule with a smooth surface, measuring 4.0 mm in size. (B) Mucosal whitening after the use of forced dehydration with induced photocoagulation (FDIP). (C) An oral mucosa break that did not epithelialize within a week (ulcer) and the central hardened exterior part of the ulcer (crust). (D) Excellent clinical healing of the lesion after one FDIP session at 24-month follow-up.

Data on age, sex, and self-reported skin color of the patient, symptoms, anatomical location, clinical appearance, size, and color of the lesion were collected. The anatomical topography of the lesions was also considered as follows: gingival/alveolar ridge, buccal mucosa, tongue, and upper and lower lip. The size of the OVA, corresponding to the largest diameter of the lesion, was measured with a millimeter ruler in a single plane (Figure 1A).

Blood profile exams (i.e., complete blood count, coagulogram, and international normalized ratio) were requested for all patients. The following outcomes were evaluated: edema, bleeding, pain, blister, ulcer, crust, scar, use of analgesics/anti-inflammatories, time to clinical healing, patient satisfaction, number of applications of FDIP, and recurrence. Before the sessions, patients were instructed about oral hygiene, care for the treated area during the healing process, observation of possible adverse effects, and use of analgesics/anti-inflammatory only when necessary.

Edema was defined as local swelling due to accumulation of fluid in the tissues, while blister was characterized as a circumscribed elevation containing more than 3.0 mm^3^ of fluid inside. Bleeding corresponded to the duration of hemorrhage. Also, ulceration was defined as a break in the oral mucosa that did not epithelialize within two weeks or where a submucosal or muscular layer was seen after detaching the necrotizing tissue, whereas crust was determined as the hardest part of the exterior surface of the ulcer. A scar was a superficial or deep mark left on the oral mucosa after the injured tissue has healed.

The time to complete clinical healing was defined as the time required to determine the achievement of complete clinical disappearance of OVA. The number of applications was defined as the number of times the patient had undergone FDIP therapy. In cases that required more than one application, the new application was made with an interval of 14 days. Another form of treatment was offered to patients who showed scarring after treatment with FDIP, and surgery was performed for functional/esthetic correction.

The FDIP response (clinical healing) was determined by any change in the lesion, i.e. lesion reduction, as follows: excellent (90–100%), good (50–89%), moderate (20–49%), and poor (0–19%)^20^ 15, 20, and 30 days post-applications. Additionally, a visual analogue scale (VAS) was utilized to evaluate patients’ perception of pain.^21^ Lesion recurrence was defined within six to 12 months following the completion of the treatment, and follow-up appointments were scheduled nine and 18 months after FDIP. The patients were also invited by telephone to express their satisfaction with the treatment received and four response options were available: ‘totally dissatisfied’, ‘partially dissatisfied’, ‘partially satisfied’, and ‘totally satisfied’.

### Device information, irradiation parameters, and treatment standards

The diode laser used in the study emits high-power infrared laser light (up to 4.5 W), with a wavelength of 808 (±10) nm, and active medium of indium-gallium-arsenide (InGaAs). Additional information about the irradiation parameters used are described in Table 1. The technique for treating OVA was FDIP. This technique is considered an alternative to laser therapy. The 810–830 nm diode laser beam is poorly absorbed by water and selectively absorbed by hemoglobin. Because of this, the laser penetrates deep into the tissue to a depth of 4.0–5.0 mm. While it passes through the tissues, the laser beam generates heat and thus coagulates the tissue to a depth of about 7.0–10.0 mm. Its selective absorption by hemoglobin and the heat generated cause selective photocoagulation within blood vessels. In the FDIP technique, diode laser energy is delivered by a flexible optical fiber without contacting the tissue. The fiber tip should not be held in the same place for too long, but should be moved slowly over the lesion while the operator observes the tissue shrinkage and blanching.^10^

**Table 1:** Device information, irradiation parameters, and treatment standards used in patients with oral vascular anomalies

Device information	Source
Manufacturer	DMC Equipamentos, São Carlos, Brazil
Model identifier	Thera Lase Surgery
Number of emitters	1
Emitter type	Indium-Gallium-Arsenide
Spatial distribution of emitters	Elliptical
Beam delivery system	Fiberoptic (400 µm)
Irradiation parameters	Measurement method or information value source
Centre wavelength (nm)	808
Spectral bandwidth (nm)	808 ± 10
Operating mode	Pulsed
Frequency (Hz)	20
Pulse on duration (ms)	25
Pulse off duration (ms)	50
Peak radiant power (W)	1.5–3.0
Average radiant power (W)	0.75–1.5
Beam profile	Gaussian
Treatment standards	Value
Beam spot size at target (cm^2^)	0.00125
Irradiance at target (W/cm^2^)	1200–2400
Average exposure duration (s)	334 (±195)
Area irradiated (mm^2^)	2–20
Application technique	Non-contact
Number and frequency of sessions	One session fortnightly

Note: µm: micrometer; cm^2^: square centimeter; Hz: Hertz; mm^2^: square millimeter; ms: milliseconds; nm: nanometer; s: seconds; SD: standard deviation; W: watts; W/cm^2^: watts per square centimeter.

Two calibrated dentists (C.N.A.O.K. and L.V.O.S.) trained in oral medicine conducted the surgical procedures under the supervision of a senior oral and maxillofacial surgeon (L.N.S.). FDIP was applied in patients under topical and/or infiltrative local anesthesia. Local anesthesia was first attempted with topical anesthetics, and infiltrative anesthesia was performed if pain persisted during FDIP. As the laser light crosses the epithelium without apparent damage, internal dehydration of the vessel and whitening of the lesion occurred, indicating the interruption of laser application (Figure 1B).

### Data analysis

The statistical analysis was performed using the Statistical Package for the Social Sciences software (SPSS), version 23.0 (IBM Inc., New Armonk, NJ, USA). Descriptive frequency analyses were carried out for clinicodemographic data and for variables collected during and after application. Recurrence and complete clinical healing during follow-up (time to clinical healing) period was assessed using survival analysis (the Kaplan-Meier method) using the MedCalc software, version 19.2.6 (MedCalc Software bv, Ostend, Belgium).

## Results

The sample of this study consisted of 47 patients, mostly male (*n=*30/63.8%), non-white (*n=*30/63.8%), with a mean age of 57.4 (±14.9) years (range: seven to 81 years). Three patients had alterations in the blood tests, two in the international normalized ratio and one in the number of platelets, but these patients had medical authorization to initiate the treatment. The most common type of OVA was varix (*n=*43/91.5%), followed by venous malformation (*n=*3/6.4%), and hemangioma (*n=*1/2.1%). Clinically, most lesions had a nodular appearance (*n=*30/63.8%), with a mean size of 7.1 (±4.9) mm. The lower lip (*n=*22/46.8%) was the most affected site (Table 2).

**Table 2:** Clinical variables of the individuals with oral vascular anomalies (*n=*47)

Variable	*n* (%)
Sex	
Male	30 (63.8)
Female	17 (36.2)
Age (years) range; mean ± SD	7–81; 57.4 ± 14.9
Skin color	
Non-white	30 (63.8)
White	14 (29.8)
Not informed	3 (6.4)
Anatomical location	
Lower lip	22 (46.8)
Upper lip	13 (27.7)
Tongue	7 (14.9)
Buccal mucosa	4 (8.5)
Alveolar ridge	1 (2.1)
Type of vascular anomaly	
Oral varix	43 (91.5)
Venous malformation	3 (6.4)
Hemangioma	1 (2.1)
Clinical manifestation	
Nodule	30 (63.8)
Papule	17 (36.2)
Color	
Purple	47 (100)
Size (mm), range; mean ± SD	2–20; 7.1 ± 4.9

Note: mm: millimeters; SD: standard deviation.

In 43 (91.5%) patients, topical local anesthetic was used, while four (8.5%) required infiltrative local anesthesia due to persistent pain in the trans-operative period. Laser was used in pulsed mode in all cases (*n=*47/100%). The peak radiant power used in each session varied between 1.5 W and 3 W, with 1.5 W and 2 W being the most used (*n=*18/38.3% for each). The FDIP time without counting the cooling time per lesion ranged from 84 to 972 seconds (mean: 334 ± 195 seconds). The number of applications ranged from one to four sessions, with the majority requiring only one FDIP session (*n=*39/83%).

Burning (*n=*25/53.2%) was the most common trans-application symptom reported by patients. Post-application findings were edema (*n=*31/66.0%), pain (*n=*13/27.6%), blister (*n=*15/31.9%), ulcer (*n=*23/48.9%), crust (*n=*31.66%) (Figure 1C), bleeding (*n=*2/4.2%), and scar (*n=*1/2.1%) (Table 3). Three patients (6.4%) reported the need for analgesics in the first two days after FDIP. Excellent clinical healing response (90–100%) was observed in all treated patients (Figure 1D), and they also reported total satisfaction with the received therapy.

**Table 3:** Trans- and post-application variables in patients with oral vascular anomalies submitted to forced dehydration with induced photocoagulation (*n=*47)

Variables	*n* (%)		Not informed *n* (%)
	Yes	No	
Trans-application symptoms	25 (53.2)	22 (46.8)	–
Post-application symptoms and signs			
Edema	31 (66.0)	16 (34.0)	–
Pain	12 (27.6)	31 (66.0)	3 (6.4)
Blister	15 (31.9)	29 (61.7)	3 (6.4)
Ulcer	23 (48.9)	21 (44.7)	3 (6.4)
Crust	31 (66.0)	13 (27.7)	3 (6.4)
Bleeding	2 (4.2)	42 (89.4)	3 (6.4)
Scar	1 (2.1)	46 (97.9)	–
Need for analgesics	3 (6.4)	41 (87.2)	3 (6.4)

**Figure 2:**
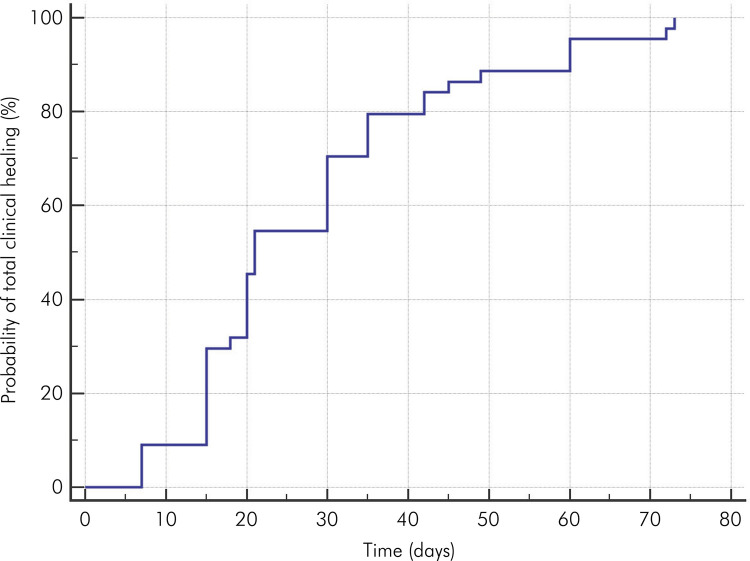
Kaplan-Meier curve for complete clinical healing of oral vascular anomalies. The highest percentages of total clinical healing were observed at 15, 20, and 30 days.

The probability of complete clinical healing of OVA was directly proportional to the increase in post-application time (days) (Figure 2). The highest percentages of complete clinical healing were observed in the 15th (*n=*9/29.5%), 20th (*n=*6/45.5%), and 30th (*n=*7/70.5%) days. The mean complete clinical healing time was 28.1 (standard error: 2.5) days. Two patients had recurrence of the lesion. Nine month post-application, the probability of recurrence was 3.4%, whereas at 18 months, this probability was 8.5%. The mean follow-up time was 17.7 (±0.4) months.

## Discussion

Data from the present study support that FDIP is safe and efficacious in the management of OVA. There was low use of local infiltrative anesthesia, low frequency of adverse effects, little need of analgesic in post-application, which was accompanied by excellent clinical healing, low recurrence rate, and most importantly, all patients were fully satisfied with the treatment. Accordingly, a previous study reported clinical healing of 98.5% of 136 cases of hemangiomas in the head and neck region submitted to FDIP and irradiated with an 810-nm high-power diode laser at 4 W and in the continuous-wave mode for 5 to 10 seconds.^10^ Success was also demonstrated in another study, since good or excellent results were obtained in 52 cases of low-flow vascular anomalies of the oral cavity after a diode laser session (with an 830 nm operating wavelength in the continuous-wave mode and 1.6 W output power). The authors reported that only six patients (10.2%) required a second diode laser application.^22^ Although not all OVA in that sample were completely removed after a single laser treatment session, the lesion volume reduction was excellent (i.e., 83% of cases) after a single session.^22^

The use of high-power diode laser in the management of OVA has increased considerably in recent years^4,7^ and shown to be effective in the treatment of superficial vascular lesions, as observed in our study and elsewhere.^7,10^ Some authors have found similar clinical results with excellent healing, even with different laser parameters.^4,7,8,10,11^ Anderson and Parrish^23^ explain the theory of photothermolysis for the treatment of vascular lesions. It is recommended to select a laser wavelength with preferential absorption by the target chromophore, an appropriate pulse duration according to the target size, and lastly a fluence that treats the target and minimizes non-specific thermal-related injury.^23^ For this purpose, the most used lasers are potassium-titanium-phosphate (KTP) (532 nm), neodymium-yttrium-aluminum-garnet (Nd:YAG) (1064 nm), pulsed dye (585 and 595 nm), argon (514 nm), carbon dioxide (CO_2_) (10,600 nm), and diode (800–980 nm).^24^ Notably, the choice of parameters, i.e. wavelength, spot size, pulse duration, and surface cooling, is critical to successful treatment.

Lasers have a number of advantages over conventional soft tissue surgery (e.g. scalpel approach), as they reduce intervention time and amount of local anesthesia required, in addition to producing hemostasis, which improves the visibility of the surgical area.^25,26^ In contrast, we have previously demonstrated that scalpel surgery had better results compared to high-power diode laser (808 nm in continuous-wave mode) in healing time of postoperative wounds of oral fibrous hyperplasia.^27^ The diode laser has important advantages, such as good coagulation properties, absence of postoperative bleeding and pain, and good wound healing.^9^ Consequently, it provides a better postoperative appearance, with less edema, bleeding, infection, and pain and thereby less need for postoperative analgesics. In our experience, in the trans-application of FDIP, approximately half of the patients felt a burning sensation, while 66% had edema and 4.2% had bleeding. Corroborating a former study,^10^ postoperative pain was reported by almost 30% of our patients, with few reporting the use of analgesics.

We believe that FDIP, a non-contact (non-invasive) laser irradiation in pulsed mode with peak radiant power mainly at 1.5 W and 2 W, did not cause excessive thermal damage to surrounding tissues. In particular, before and during sessions, the surfaces were also cooled with cold saline solution to protect the tissue surface from damage.^10^ According to Angiero et al.^10^, the heat generated by continuous-wave diode laser energy was only effective superficially and was sufficient to photocoagulate small oral hemangiomas. However, it is important to mention that, compared to continuous-wave mode, the pulsed operation mode may result in less tissue damage adjacent to the treated sites.^9^

If the lesion is not completely resolved in the first session, diode laser treatment can be repeated in the OVA lesions.^7,9,26,28-30^ Herein, five patients needed two FDIP sessions, two needed three sessions, and one had four application sessions. It is noteworthy that those patients who required more than one FDIP session exhibited lesions with an approximate size of 15.0 mm. This fact may suggest that FDIP by diode laser may be a safe and efficacious treatment for small lesions. In this context, some authors reported the impossibility of treating deep vascular lesions using the transmucosal approach,^31,32^ while others^10^ excluded patients whose vascular lesions had a diameter larger than 3.0 cm because laser therapy would require several sessions and because such large lesions should be treated using selective embolization.

Therapies for OVA continue to pose a dilemma for clinicians and oral surgeons.^11^ Several treatment options have been described for these conditions, including conventional surgery with or without adjunctive preoperative embolization, cryosurgery, and drug therapies (e.g., ethanolamine oleate and polidocanol).^5,33,34^ However, these therapeutic approaches carry a risk of side effects such as scarring, pain, and bleeding.^5^ Current advances in the use of high-power lasers allow oral health professionals to provide effective treatments with minimal side effects.^30,31,35,36^ Nonetheless, it is important to emphasize the high cost of the laser device compared to alternative or conventional therapies and the steep learning curve for the practitioner to operate the device properly.^7^ Reports of accidental laser/light eye injuries among operators and patients have also been documented, even when of protective eyewear;^37^ these are certainly the main limiting factors for this therapeutic modality. An additional drawback of diode lasers compared to other lasers is that the maximum power output is 150 W.^9^ CO_2_ lasers are known to interact much better with water than near-infrared diode lasers.^38^ Conversely, diode lasers using fiber optics allow better access to some areas of the oral cavity (e.g. posterior areas) and offer much more accessibility than CO_2_ handpieces.^38^

This study has shortcoming inherent to case series designs and therefore should be acknowledged. The small sample size is likely to incorporate unforeseen bias, and statistical results should be interpreted with caution. In addition, efforts were made to collect information on patient outcome, but some individuals did not attend the follow-up appointment. Further studies with a large sample size, a longer follow-up, and FDIP application with different doses/protocols are recommended, including complete descriptions of the device and parameters employed.

## Conclusion

In summary, FDIP by diode laser, when used properly, is an efficacious and safe treatment approach for OVA. Our results reveal that this is a promising alternative therapy that can promote clinical healing, most of the time, with in one session and with few adverse effects during and after application.
