# Erratum

**DOI:** 10.1590/1807-3107bor-2024.vol38.0025.erratum

**Published:** 2024-07-22

In article Experience with 808-nm diode laser in the treatment of 47 cases of oral vascular anomalies, with DOI number 10.1590/1807-3107bor-2024.vol38.0025, published in the journal Brazilian Oral Research, v. 38, e025, 2024, on

**p. 1**

Where is read: José Alcides Almeida de ARRUDA

Should read: José Alcides Almeida DE ARRUDA

**p. 3, 5, 7, 9**

Where is read: de Arruda JAA

Should read: De Arruda JAA

